# Disability Adjusted Life Years due to Ischaemic Stroke Preventable by Real-Time Stroke Detection—A Cost-Utility Analysis of Hypothetical Stroke Detection Devices

**DOI:** 10.3389/fneur.2018.00814

**Published:** 2018-10-01

**Authors:** Ludwig Schlemm

**Affiliations:** ^1^Department of Neurology, Charité–Universitätsmedizin Berlin, Berlin, Germany; ^2^Center for Stroke Research Berlin, Charité–Universitätsmedizin, Berlin, Germany; ^3^Berlin Institute of Health, Berlin, Germany; ^4^Department of Health Policy, London School of Economics and Political Science, London, United Kingdom

**Keywords:** ischaemic stroke, endovascular treatment, thrombectomy, thrombolysis, prehospital triage, mathematical modeling, cost-effectiveness analysis, health economics

## Abstract

**Background:** Ischaemic stroke remains a significant contributor to permanent disability world-wide. Therapeutic interventions for acute ischaemic stroke (AIS) are available, but need to be administered early after symptom onset in order to be effective. Currently, one of the main factors responsible for poor clinical outcome is an unnecessary long time between symptom onset and arrival at a hospital (pre-hospital delay). In the future, technological devices with the capability of real-time detection of AIS may become available. The health economic implications of such devices have not been explored.

**Methods:** We developed a novel probabilistic model to estimate the maximally allowable annual costs of different hypothetical real-time AIS detection devices in different populations given currently accepted willingness-to-pay thresholds. Distributions of model parameters were extracted from the literature. Effectiveness of the intervention was quantified as reduction in disability-adjusted life-years associated with faster access to thrombolysis and mechanical thrombectomy. Incremental costs were calculated from a societal perspective including acute treatment costs and long-term costs for nursing care, home help, and loss of production. The impact of individual model parameters was explored in one-way and multi-way sensitivity analyses.

**Results:** The model yields significantly shorter prehospital delays and a higher proportion of acute ischaemic patients that fulfill the time-based eligibility criteria for thrombolysis or mechanical thrombectomy in the scenario with a real-time stroke detection device as compared to the control scenario. Depending on the sociodemographic and geographic characteristics of the study population and operating characteristics of the device, the maximally allowable annual cost for the device to operate in a cost-effective manner assuming a willingness-to-pay threshold of GBP 30.000 ranges from GBP 22.00 to GBP 9,952.00. Considering the results of multiway sensitivity analyses, the upper bound increases to GBP 29,449.10 in the subgroup of young patients with a very high annual risk of ischaemic stroke (50 years/20% annual risk).

**Conclusion:** Data from probabilistic modeling suggest that real-time AIS detection devices can be expected to be cost-effective only for a small group of highly selected individuals.

## Introduction

### Background

Ischaemic stroke is among the leading causes for permanent disability world-wide (1). Treatment options for patients with acute ischaemic stroke (AIS) include intravenous thrombolysis and mechanical thrombectomy (MT) (2, 3). These therapeutic interventions are most effective when initiated early (4), with previous studies having shown a reduction in long-term disability of up to 12.5 disability-adjusted days for each minute that treatment is initiated earlier (5, 6). In the past years, efforts to develop efficient intra-hospital processes and high-priority standardized pathways for hyper-acute stroke care have led to significant reductions in intra-hospital delays (7–10). However, prehospital delays, i.e., time from symptom onset to arrival at the hospital, have remained largely unchanged and are therefore seen as a potential opportunity for further improvement in the provision of timely access to effective treatment for patients with AIS (11). Prehospital delays result mainly from two different factors: (1) patients and/or bystanders do not recognize the urgency of their symptoms and do not present to hospitals immediately (11); and (2) strokes happen at night and patients recognize their symptoms only when waking up (12). In addition, patients may be ineligible for acute treatment if the time of symptom onset is unknown (either due to stroke-related symptoms such as aphasia or due to strokes occurring during the night). Efforts have been undertaken to reduce the impact of these contributing factors, namely the implementation of education campaigns aiming to improve recognition of stroke symptoms (13, 14), and use of advanced magnetic resonance imaging protocols as “tissue clocks” to determine the time of symptom onset (15). In the domain of cardiac electrophysiology, implantable cardioverter-defibrillators are available to constantly monitor high-risk patients for the occurrence of potentially lethal cardiac arrhythmias and to administer treatment immediately (16, 17). Similar technologies for the real-time detection of AIS do not currently exist but would have the potential to significantly reduce onset-to-treatment times and improve outcome for patients with ischaemic stroke. The health economic implications associated with the availability and use of such real-time stroke detection devices have not been explored. Since the likelihood of market access and reimbursement of new medical devices is closely linked to their cost-effectiveness, a health economic assessment of the potential impact of real-time stroke detection devices should precede the decision about potential financial investments in their development.

### Objective of the current study

In the current study, we aim to assess whether real-time stroke detection devices could be expected to operate in a cost-effective manner. For this, we derive maximally allowable total annual costs for different hypothetical devices in different populations given currently accepted willingness-to-pay thresholds.

## Methods

### Overview

We built a conditional probabilistic model to estimate the number of disability-adjusted life-years (DALYs) that could be prevented by real-time pre-hospital detection of patients with AIS. For this, we compared a scenario in which a real-time AIS detection device is available, with a scenario without such a device (control scenario, current standard of care). We calculated expected incremental costs and incremental benefits, expressed as estimated reduction in DALYs associated with faster access to thrombolysis and MT. Assuming a range of commonly accepted societal willingness-to-pay-thresholds, we obtained estimates for the maximally allowable annual cost of a real-time AIS detection device (MAACD). Parameters of the model were chosen according to empirical distributions extracted from the literature. Since MAACD is expected to depend on demographic (age, sex), clinical (annual risk of ischaemic stroke), and socio-geographic characteristics (prehospital delay due to poor education with regards to symptoms of AIS; expected time the patients was last seen well before the incident [TLSW]/is found after the incident [TFAI]; and expected transfer times and treatment time metrics at the hospital [urbanictiy]; Table 1) of the population the device is intended for, results are reported according to different combinations of these individual characteristics. Only patients without contraindications to thrombolysis and MT and without known conditions predisposing to the development of symptoms similar to those of AIS (stroke mimics; e.g., epilepsy, migraine, multiple sclerosis) were considered as potential candidates in our model.

**Table 1:** Parameters representing individual characteristics and living circumstances used in the model.

**Parameter**	**Structure**	**Base case parameters**	**Range**	**References/details**
Age, a	Single parameter	50–90 years	–	–
Sex, s	Single parameter	Male, Female	–	–
Absolute annual risk of ischaemic stroke, r	Single parameter	0.01–0.2	–	–
Urbanicity, U(TD1, DTN, NTG)	Set of parameters TD1: time to nearest PSC DTN: door-to-needle time at nearest PSC NTG: needle-to-groin time	TD1: 30 min DTN: 45 min NTG: 90 min	Urban environment: TD1: 15 min; DTN: 30 min; NTG: 60 min Rural environment: TD1: 60 min; DTN: 60 min; NTG: 120 min	Used to define socio-geographic scenarios I – VIII
Delay due to poor education with regards to stroke symptoms, ED	$ED\left(NIHSS\right)=60a\times \left(1-\frac{1}{1+e^{\frac{b-NIHSS}{c}}}\right)$	a = 3; b = 2; c = 3;	Poorer education: a = 1; b = 0; c = 2; Better education: a = 12; b = 2; c = 2;	Used to define socio-geographic scenarios I – VIII; Figure S1
Expected time last seen well before ischaemic stroke incident / expected time found after ischaemic stroke incident, TLSW / TFAI	Single parameter	30 min	More frequent visits: 10 min Less frequent visits: 180 min	Used to define socio-geographic scenarios I – VIII

*NIHSS stands for National Institutes of Health Stroke Scale*.

### Description of the model

**Figure 1:**
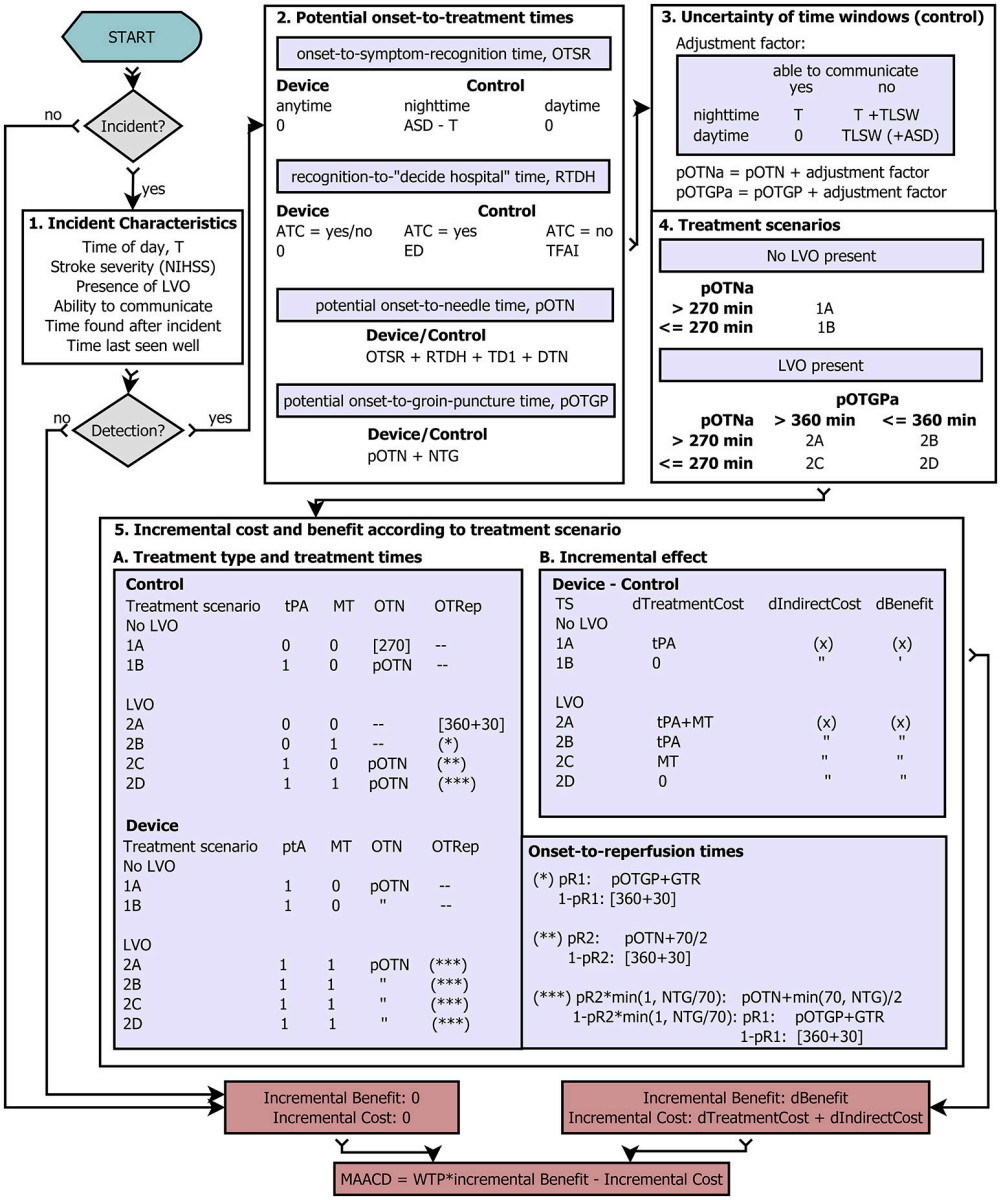
Flow diagram. Graphic representation of the probabilistic conditional model. For a detailed description, see main text. (x) represents multi-parametric functions of age, sex, NIHSS, and reduction in symptom onset-to-needle/symptom onset-to-reperfusion times. If patients do not fulfill the time-based eligibility criteria for thrombolysis or mechanical thrombectomy, or if no reperfusion is achieved, the upper end of the respective treatment time window at which the administration of the respective treatment is associated is with no benefit used for the calculation of the reduction in time-to-treatment (indicated by brackets []). **General Abbreviations:** T stand for the time of day at which the acute ischaemic stroke incident occurs, NIHSS for National Institutes of Health Stroke Scale, LVO for large vessel occlusion, ASD for average sleep duration (night time), ATC for ability to communicate (i.e., call emergency medical services and/or give information about symptom onset), tPA for intravenous thrombolysis with tissue-type plasminogen activator, MT for mechanical thrombectomy, TS for treatment scenario, pR1 for the probability of reperfusion of large vessel occlusion during mechanical thrombectomy, pR2 for the Probability of reperfusion of large vessel occlusion 70 min after intravenous thrombolysis, WTP for willingness-to-pay threshold, and MAACD for the maximally allowable annual cost of the device. **Time interval abbreviations:** OTSR stands for symptom onset-to-recognition time, RTDH for symptom recognition-to-(decision to go to hospital)-time, ED for prehospital delay between recognition of symptoms and the decision to go to a thrombolysis-ready hospital attributable to poor education about stroke symptoms, TFAI for the average time a patient is found after an incident, pOTN for potential symptom onset-to-needle time, pOTGP for potential symptom onset-to-groin puncture time, pOTNa and pOTGPa for potential symptom onset-to-needle time and potential symptom onset-to-groin puncture time, respectively, after adjustment for the uncertainty of time windows, TD1 for travel time to the nearest thrombolysis-ready hospital, DTN for door-to-needle time, NTG for needle-to-groin puncture time, TLSW for the average time a patient was last seen well before the incident, OTRep for symptom onset-to-reperfusion time.

A graphic representation of the model is presented in Figure 1. For a detailed exposition of the parameters used in the model, see Tables 1–6. The process starts in the upper left hand corner. First, it is determined whether an ischaemic stroke occurs in a given year (“Incident?”) according to the *a priori* defined annual risk of ischaemic stroke (range 1–20%). If no ischaemic stroke occurs, the incremental benefit of the stroke detection device is 0 DALYs, and the incremental cost (excluding the device cost) is GBP 0.00. If an ischaemic stroke occurs, the model determines the incident characteristics (Figure 1, Box 1): time of day, stroke severity, presence of large vessel occlusion (LVO), whether the patient is able to communicate (ATC), time delay before the patient would be found after the incident (TFAI), and length of time since the patient was last seen well before the incident (TLSW). Next, it is determined whether a real-time AIS detection device with a certain assumed mode of operation and sensitivity detects the ischaemic stroke incident. If the device fails to detect the incident, the incremental benefit and incremental cost (excluding the annual cost of the device), are 0 DALYs and GBP 0.00, respectively. Otherwise, for both the scenario with a real-time AIS detection device and the control scenario, potential onset-to-treatment times are calculated based on the time of the day, the ability to communicate, the likelihood of the patient to recognize the need for rapid medical attention (quantified as expected delay due to poor education with regard to stroke symptoms [ED]), expected time delay before the patient is found after the incident [TFAI], and the stroke care infrastructure in the patient's environment (urbanicity: transport time, door-to-needle time, needle-to-groin time; Figure 1, Box 2). Next, for the control scenario, potential onset-to-treatment times are adjusted to account for the uncertainty of time windows, which can results from either the stroke incidence occurring during the night, or the patient being unable to communicate, or both (Figure 1, Box 3). According to these adjusted onset-to-treatment times (which in clinical praxis form the basis of treatment decisions if no other means of determining the time of symptom-onset such as magnetic resonance imaging is available) and the vessel status, the model categorizes the patients in the control scenario in one of six treatment scenarios defined by the time-based eligibility criteria for thrombolysis and MT (Figure 1, Box 4; note that the patients in the scenario with a real-time AIS detection device always fulfill the time-based eligibility criteria for both thrombolysis and MT). Next, the potential onset-to-treatment times, the vessel status, and the treatment scenario, i.e., the time-based eligibility for thrombolysis and/or MT, are used to determine the type of acute treatment administered (i.v. thrombolysis, MT, or both) and the effective treatment times. For patients without LVO, the relevant treatment time metric is symptom onset-to-needle time; for patients with LVO, symptom-onset-to-reperfusion time. Regarding the latter, reperfusion of LVO can either be achieved by thrombolysis alone, or through successful MT. Last, the incremental costs and benefits are determined by comparing costs and benefits between the control scenario and the scenario with a detection device. Based on these incremental effects, the maximally allowable annual cost of a real-time AIS detection device is calculated. For each set of input parameters (age, sex, risk, and socio-geographic scenario), the simulation was repeated n = 20.000 times, and the resulting mean and standard deviation of the MAACD were calculated. The time frame for the intervention was one single year because data on the evolution of annual risk of ischaemic stroke over time and on the relationship between risk for ischaemic stroke and mortality were not available. Consequences of the intervention were considered over a time frame of 25 years for costs and over the whole lifetime for benefits.

**Table 2:** Clinical parameters used in the model.

**Parameter**	**Structure**	**Base case parameters**	**Range**	**References/details**
Time of ischaemic stroke occurrence, T	Probability distribution (uniform) $p\left(T\right)=\frac{1}{24\times 60}$	–	–	Figure S1
Symptom severity, NIHSS	Probability distribution $p\left(NIHSS\right)=\frac{p1\times NIHSS+p2}{NIHSS^{2}+q1\times NIHSS+q2}$	p1 = 0.068; p2 = 1.137; q1 = −2.288; q2 = 10.02	More severe strokes: p1 = 1; p2 = 1; q1 = −1; q2 = 4 Less severe strokes: p1 = 0.02; p2 = 1; q1 = − 0.3; q2 = 5	(18) Figure S2
Ability to communicate, ATC	Probability function of NIHSS $P_{ATC=1}\left(NIHSS\right)=1-\frac{1}{1+e^{\frac{a-NIHSS}{b}}}$ *P*_*ATC* = 0_(*NIHSS*) = 1−*P*_*ATC* = 1_(*NIHSS*)	a = 15; b = 3	Less able to communicate: a = 10; b = 2.5 More able to communicate: a = 20; b = 2	Reasonable assumption. Wide range for sensitivity analyses. Figure S3
Presence of large vessel occlusion, LVO	Probability function of NIHSS $P_{LVO=1}\left(NIHSS\right)=\frac{a}{1+e^{\frac{b-NIHSS}{c}}}$ *P*_*LVO* = 0_(*NIHSS*) = 1−*P*_*LVO* = 1_(*NIHSS*)	a = 0.957; b = 14.12; c = 5.551	More frequent LVO: a = 0.985; b = 9.359; c = 3.718 Less frequent LVO: a = 0.941; b = 18.61; c = 5.62	(19) (20) (21) Figure S4

*NIHSS stands for National Institutes of Health Stroke Scale, ATC for ability to communicate (defined as inability to contact emergency medical personnel independently and to give information about symptom onset due to stroke symptom severity [e.g., aphasia, motor impairment, reduced level of consciousness]), and LVO for large vessel occlusion*.

**Table 3:** Treatment related parameters used in the model.

**Parameter**	**Structure**	**Base case parameters**	**Range**	**References/details**
Treatment time window for intravenous thrombolysis (maximal symptom onset-to needle time)	Single parameter	270 min	–	(2)
Treatment time window for mechanical thrombectomy (maximal symptom onset-to-groin puncture time)	Single parameter	360 min	–	(3)
Treatment effect: reduction in DALYs per minute faster treatment, TE	Δ*DALY = f(sex,age,NIHSS,LVO)*	Point estimates fitted using a locally weighted smoothing linear regression (span 0.2)	Upper and lower 95% prediction interval	(5, 6)
Probability of reperfusion of LVO 70 min after thrombolysis, pR2	Single parameter	0.1; linear adjustment if time from begin of thrombolysis to groin puncture (needle-to-groin [NTG]) is <70 min: pR2^*^min(1, NTG/70)	–	(22) (22)
Probability of reperfusion of LVO during mechanical thrombectomy, pR1	Single parameter	0.8	–	(22)
Groin puncture-to-reperfusion time, GPR	Single parameter	30 min	–	(22)

*DALY stands for disability-adjusted life-year, TE for treatment effect, NIHSS for National Institutes of Health Stroke Scale, LVO for large vessel occlusion, and NTG for needle-to-groin time*.

**Table 4:** Device related parameters used in the model.

**Parameter**	**Structure**	**Base case parameters**	**Range**	**References/details**
Mode of operation, M	Classification with regard to possible modes of operation	M3: Detection of all acute ischaemic strokes during day and night	M1: detection limited to ischaemic stroke due to LVO M2: detection limited to ischaemic strokes occurring during the day	–
Device sensitivity, S	Sensitivity to detect acute ischaemic stroke	S3: 75%	S1: 50% S2 100%	–

**Table 5:** Cost parameters used in the model.

**Parameter**	**Structure**	**Base case value/parameters**	**Range**	**References/details**
Intravenous thrombolysis	Single parameter	GBP 1,926.13 (USD 2,953.59 in 2015; exchange rate GBP 1 = USD 1.5388)	–	(23)
Mechanical thrombectomy	Single parameter	GBP 9,050.98 (USD 13,803.04 in 2015; exchange rate GBP 1 = USD 1.5388)	–	(23)
Reduction in indirect long-term costs after ischaemic stroke due to home help, nursing care, and loss of production	Δ*IndirectCosts = f(sex,age,NIHSS,LVO)*	Age-specific data on indirect cost savings per minute faster treatment was anchored to reductions in DALYs (median NIHSS = 14) and transformed to other NIHSS values assuming proportionality of reductions DALYs and indirect cost savings.	–	(24) (6) Figure S5
Willingness-to-pay threshold	Single parameter	GBP 30,000.00	GBP 20.000,00, GBP 40.000,00	(25)

*NIHSS stands for National Institutes of Health Stroke Scale, LVO for large vessel occlusion, and DALY for disability-adjusted life-year*.

**Table 6:** Miscellaneous parameters used in the model.

**Parameter**	**Structure**	**Base case parameters**	**Range**	**References/ details**
Average sleep duration, ASD	Single parameter	7.5^*^60 min	Less sleep: 6^*^60 min More sleep: 9^*^60 min	(26) Figure S6

### Effectiveness estimate

Estimated number of DALYs preventable by a real-time ischaemic stroke detection device are calculated based on data from Meretoja et al. (5, 6) The authors used logistic regression models to estimate the improvement in functional outcome and subsequently the reduction in DALYs associated with a 1-min decrease in time to thrombolysis for patients without LVO, and in time to reperfusion for patients with LVO. For thrombolysis, their results were based on a published meta-analysis of randomized controlled trials (27). For MT, the authors used data on the relationship between time to reperfusion and treatment effect observed in a single large randomized controlled trial (28). For patients with LVO, we therefore adopted a physiological perspective focusing on reperfusion instead of time-to-groin puncture. DALYs were calculated according to current WHO guidelines without age-weighing or discounting (1). We used the age-, sex-, vessel status- and stroke severity-specific estimates presented by Meretoja et al. to create multi-parametric fits (locally weighted smoothing linear regression with a span of 0.2) for the age range 50–90 years and the stroke severity range 0–42 points on the NIHSS scale. Due to limited availability of data, stroke severities >25 points were mapped to corresponding values of NIHSS = 25.

### Cost estimate

For our analysis, we adopted a societal point of view and considered three different types of cost in our analyses (Table 5). First, direct in-hospital costs associated with the administration of thrombolysis and/or MT (23). Additional costs, such as day costs in the neurological department and diagnostic procedures, were not considered as these would not generally be affected by the use of a real-time AIS detection device. Potential cost savings attributable to reduced length of stay due to better clinical outcome as a consequence of faster treatment were not considered due to lack of data. Second, long term costs for home help, nursing home care, and loss of production. Average age-specific estimates for these costs were extracted from Steen Carlsson et al. (24) and, assuming a proportional relationship with DALYs, transformed to account for different stroke severities and reductions in onset-to-treatment times using the multi-parametric functions derived from Meretoja et al. (5) and Meretoja et al. (6) Third, the annual cost of the real-time AIS detection device was considered. Here, we did not differentiate between costs to set up the device and annual running costs, because the time-frame of the intervention was limited to 1 year. False positive detections (i.e., visits to the emergency department with a final diagnosis other than AIS [stroke mimic or haemorrhagic stroke]) were not considered in the calculation of incremental costs, because these patients should not receive thrombolysis or MT after careful examination and history taking by a neurologist and neuroimaging, and because, given our exclusion criteria of known medical conditions leading to stroke mimics, such patients should receive an urgent neurological diagnostic workup in the control scenario also. All costs are expressed as GBP of the year 2018 with an inflation rate of 3%. Official exchange rates from the Bank of England were used for the conversion of foreign currencies.

### Main outcome

The main outcome of the analyses was the MAACD for individuals with different demographic, clinical, and socio-geographic characteristics given a willingness-to-pay threshold of GBP 30,000 (25). All simulations and analyses were performed in MATLAB (29). For the sake of clarity of presentation, results for men and women were aggregated through arithmetic averaging; however, sex-specific results were also calculated and are available upon request.

### Analysis plan

First, we present results using a set of base-case scenario parameters. We then examine the influence of different modes of detection of the device and different sensitivities. Next, the impact of individual model parameters on outcome is explored in one-way and multiway sensitivity analyses. Last, we present results for populations with exemplary demographic and clinical characteristics with uncertainty quantified by 95% confidence intervals.

## Results

### Impact of real-time stroke detection

**Figure 2:**
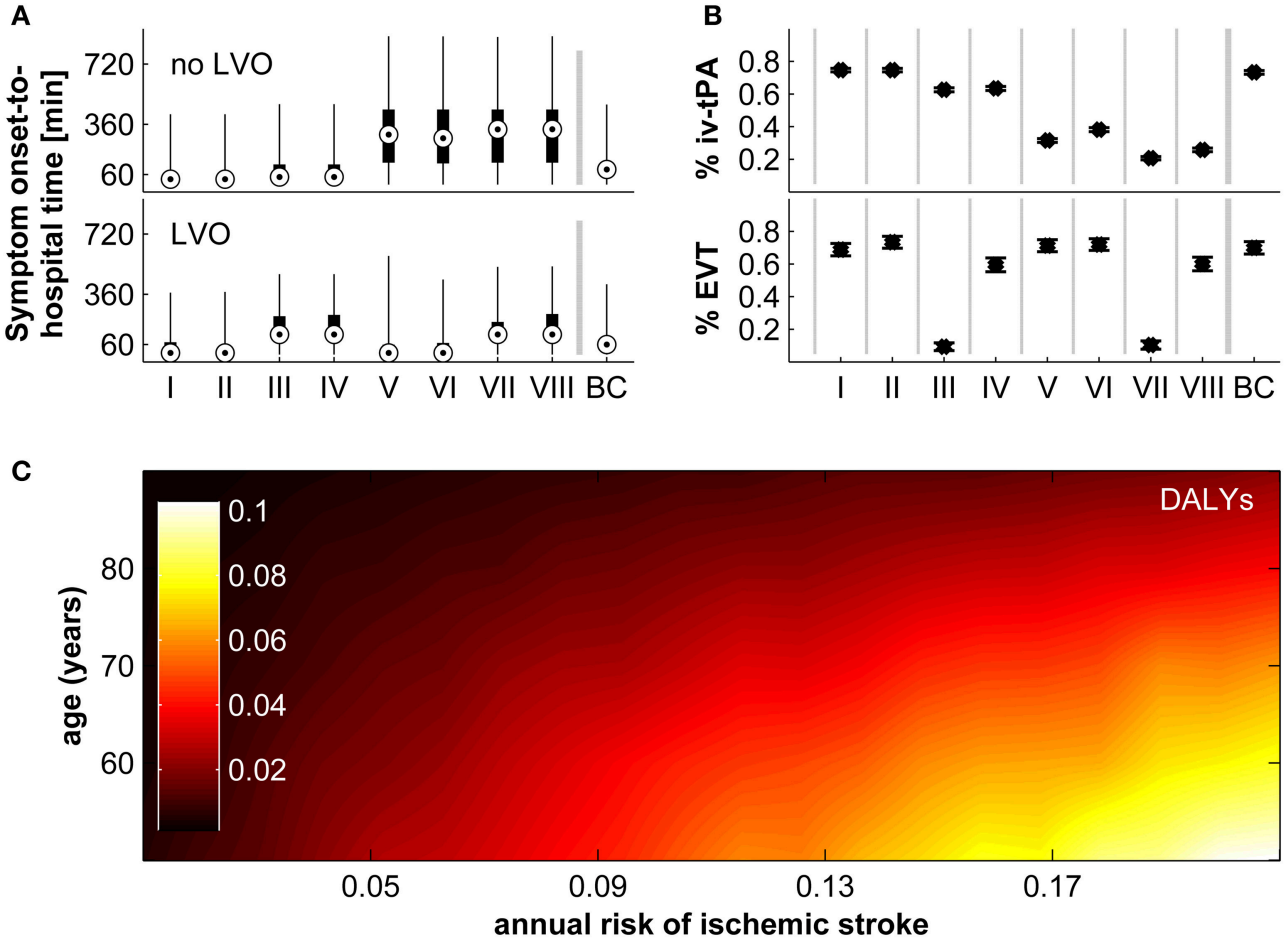
Impact of real-time stroke detection. **(A)** Reduction of symptom onset-to-hospital time for patients with and without large vessel occlusion (LVO) in socio-geographic scenarios I–VIII and in the base-case scenario (BC). Shown are the median (circles), interquartile range (black boxes), and full range. **(B)** Proportion of all patients with acute ischaemic stroke fulfilling time-based eligibility criteria for thrombolysis (top), and proportion of all patients with acute ischaemic stroke with LVO fulfilling the time-based eligibility criteria for mechanical thrombectomy (bottom). Shown are the median (circles), interquartile range (black boxes), and full range. **(C)** Disability adjusted life-years (DALYs) preventable by real-time stroke detection according to age and annual risk for ischaemic stroke assuming parameters of the base-case scenario. For a definition of socio-geographic scenarios, see main text and Table 7.

The beneficial effect of real-time detection of AIS and immediate notification of emergency medical services is achieved through reduced prehospital delays, less uncertainty regarding the true time of symptom onset, and a higher probability to fulfill the time-based eligibility criteria for thrombolysis and MT. First, we calculated the estimated reduction in time from symptom onset-to-hospital arrival in our model according to vessel status and socio-geographic scenario (I–VIII; Figure 2A). For patients without LVO, the reduction in time from symptom onset-to-hospital arrival is most strongly influenced by delay due to poor education about the urgency of stroke symptoms and the need for immediate treatment (ED). For patients with LVO, the main difference is observed between socio-geographic scenarios I, II, V, VI on one hand, and III, IV, VII, VIII on the other hand, which implies a strong influence of the parameter “time found after the incident/time last seen well before the incident” (TFAI/TLSW). This difference in the influence of model parameters between the group of patients with and without LVO is explained by the observation that patients with LVO are more likely to have more severe stroke symptoms and are therefore more likely to depend on being found by others, while patients without LVO are more likely to have less severe stroke symptoms, in which case the prehospital delay depends more strongly on the patients' own educational status. Next, we modeled the percentage of all AIS patients fulfilling the time-based eligibility criteria for thrombolysis and the percentage of all AIS patients with LVO fulfilling the time-based eligibility criteria MT in the control scenario without a real-time AIS detection device (Figure 2B; note that in the scenario with a real-time AIS detection device, all patients would, by definition, fulfill the time-based eligibility criteria for thrombolysis and MT). With regards to the eligibility for thrombolysis, the difference between the control scenario and the intervention scenario is most pronounced in socio-geographic scenarios V–VIII, again corresponding to poorer education about stroke symptoms. With regards to eligibility for MT, the most pronounced reductions are observed in socio-geographic scenarios III and VIII, corresponding to the combination of a rural geographic environment and a longer expected time the patient is found after the incident/last seen well before the incident (TFAI/TLSW).

**Table 7:** Definition of socio-geographic scenarios I–VIII.

**Frequency of visits (quantified by TLSW/TFAI)**	**Urbanicity**	**Education with regards to stroke symptoms**	
		**Better**	**Worse**
More frequent	Urban	I	V
	Rural	II	VI
Less frequent	Urban	III	VII
	Rural	IV	VIII

*For parameter values, see Table 1. TLSW stands for the expected time the patient was last seen well before the ischaemic stroke incident; TFAI for the expected time the patient would be found after the ischaemic stroke incident*.

The longer delay from symptom onset to the administration of thrombolysis or MT, or indeed the ineligibility for these treatments due to exceeded treatment time windows, translates directly into worse functional outcome. In Figure 2C we display the expected reduction in DALYs achievable by real-time AIS detection for the base-case scenario according to age and annual ischaemic stroke risk. The estimated reduction in long-term disability ranges from 0.00 to 0.10 DALYs and shows a strong correlation with both age and annual ischaemic stroke risk.

### Maximally allowable annual cost of the device in the base-case scenario

**Figure 3:**
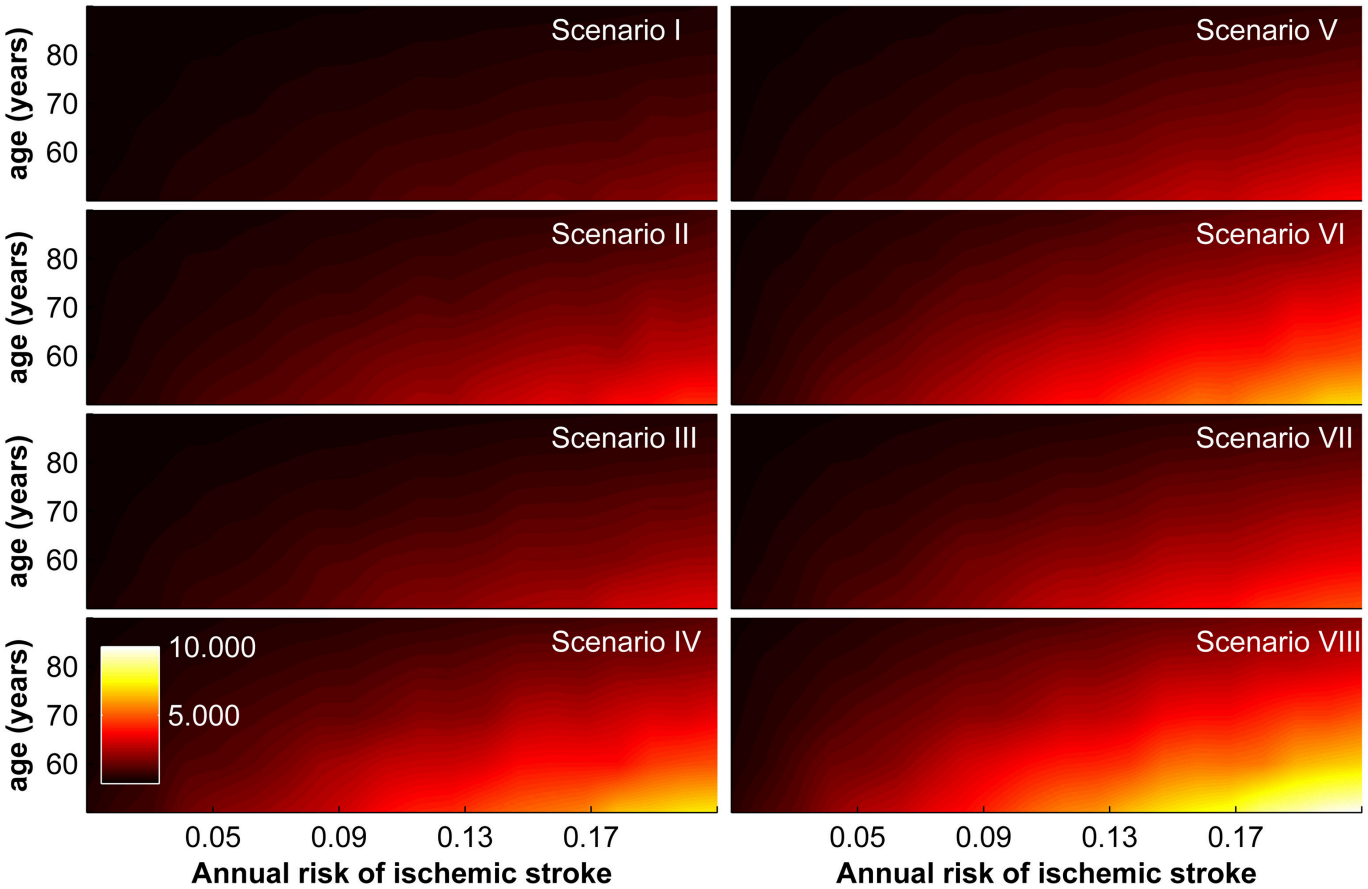
Maximally allowable annual cost of device according to age and annual risk of ischaemic stroke for socio-geographic scenarios I–VIII. For a definition of socio-geographic scenarios, see main text and Table 7.

Combining these estimated reductions in DALYs with incremental costs and assuming a willingness-to-pay threshold of GBP 30,000, we obtain the maximally allowable annual cost of the device for the base-case scenario. It ranges from GBP 22.00–9,952.00 and is again strongly correlated with age, annual risk of ischaemic stroke, and socio-geographic scenario (Figure 3).

### Impact of assuming different modes of operation

**Figure 4:**
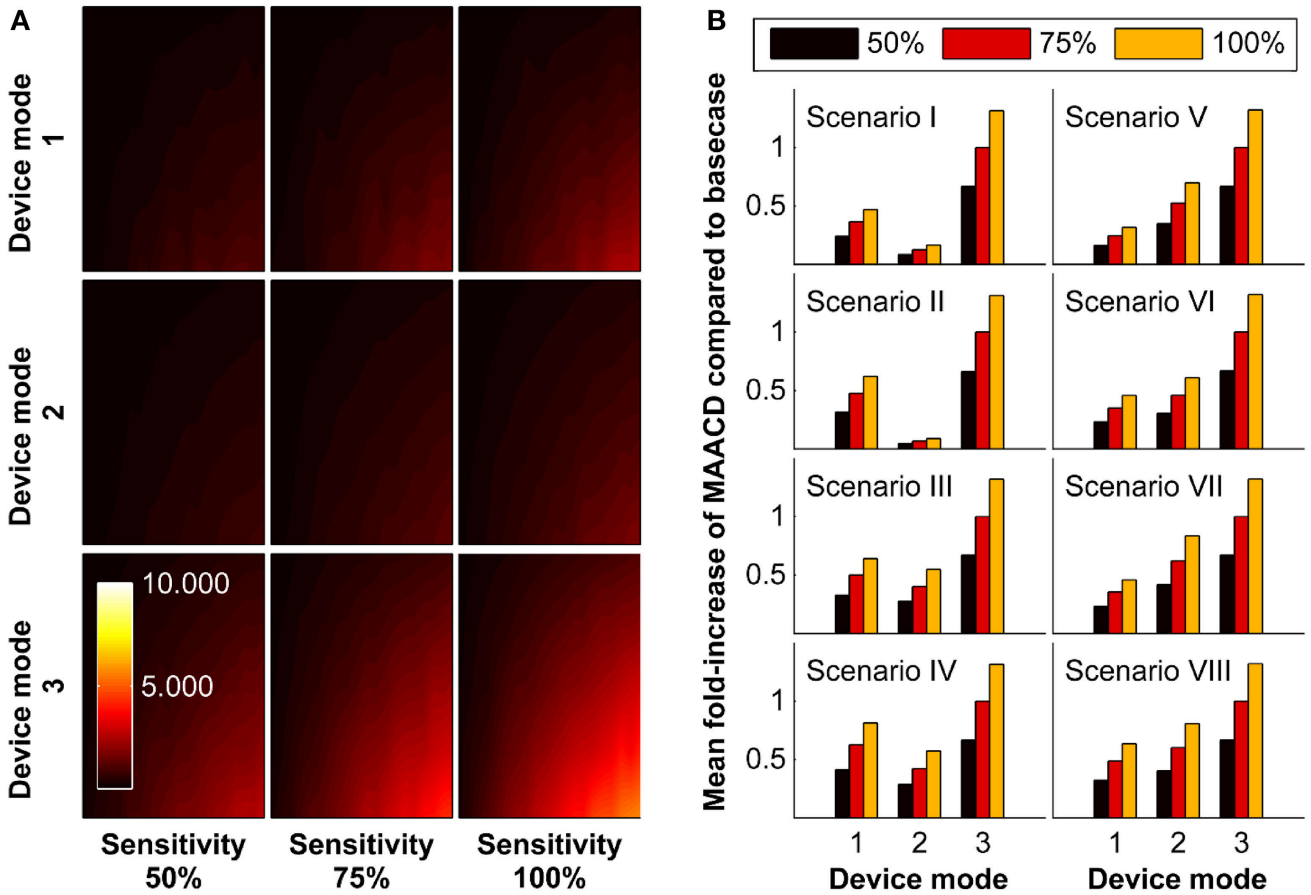
Impact of mode of operation of the device and sensitivity of stroke detection on maximally allowable annual cost of device (MAACD) according to age and annual risk of ischaemic stroke. **(A)** MAACD according to age (vertical axes, 50–90 years) and annual risk of ischaemic stroke (horizontal axis, 1–20%) for different combinations of mode of operation of the device (M1, M2, M3) and sensitivity for stroke detection (50%, 75%, 100%) in the base-case socio-geographic scenario. **(B)** Mean fold-increase of MAACD associated with different combinations of mode of operation of the device (M1, M2, M3) and sensitivity for stroke detection (50%, 75%, 100%) compared to the base-case scenario (mode M3/sensitivity 75%) in socio-geographic scenarios I–VIII. MAACD ratios are not influenced by age or annual risk of ischaemic stroke (data not shown). For a definition of socio-geographic scenarios and modes of operation of the device, see main text and Tables 4, 7.

In the base-case scenario, we assumed a real-time AIS detection device that is able to detect ischaemic stroke irrespective of the presence or absence of an associated LVO and irrespective of time of day (night time or day time; mode M3) with a sensitivity of 75%. The impact of assuming different modes of operation (detection limited to incidents due to LVO [mode M1], detection limited to day time when the patients is active [mode M2]) and different sensitivities (50%, 100%) is shown in Figure 4. In all socio-geographic scenarios, devices operating in mode M1 and M2 have a consistently lower MAACD than devices operating in mode M3. For devices with a mode of operation M3, increasing the sensitivity of stroke detection from 75 to 100% is associated with an increase in the MAACD of 31.2–32.2% (Figure 4B).

### One-way and multi-way sensitivity analyses of model parameters

**Figure 5:**
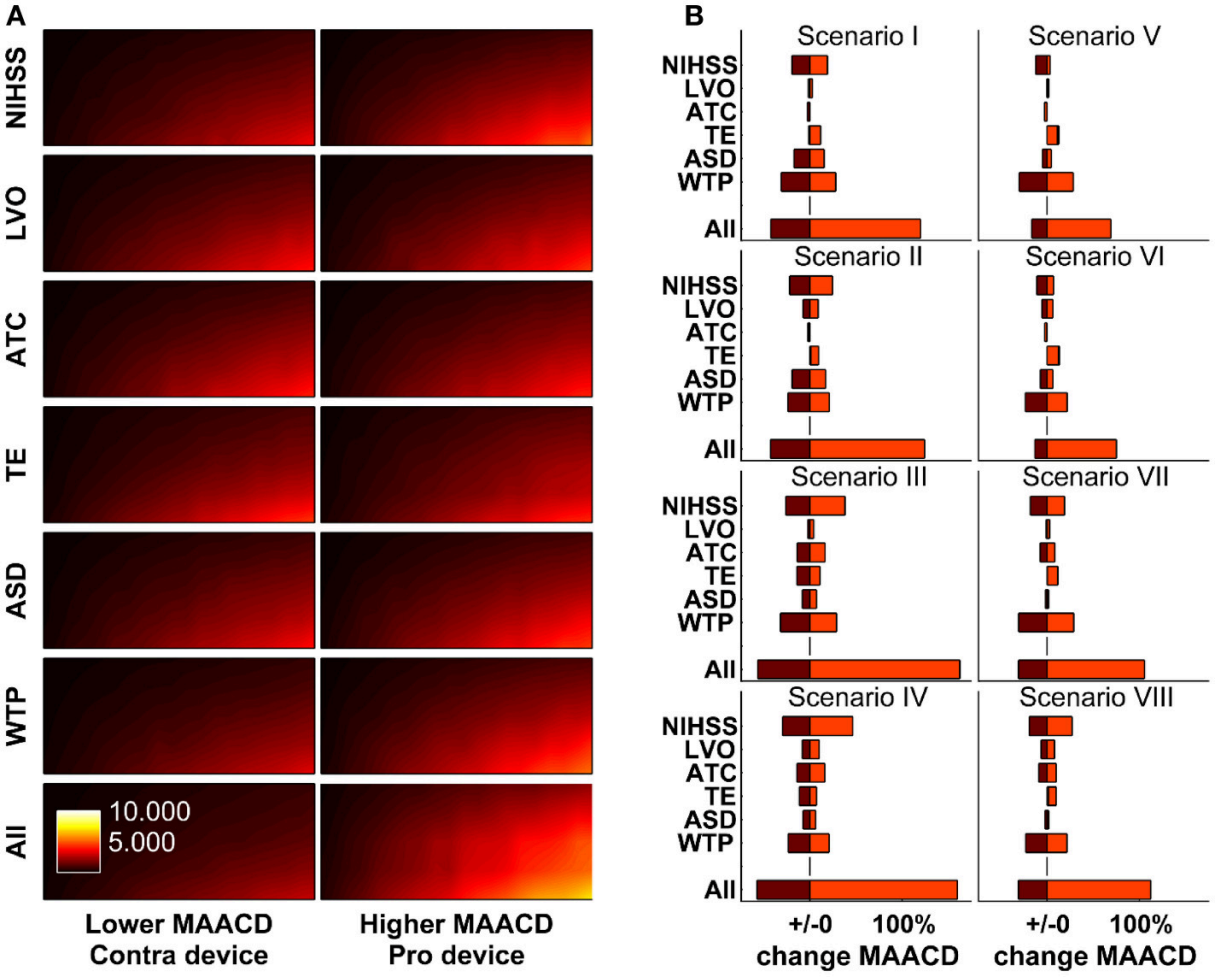
One-way and multi-way sensitivity analyses. Impact of changes of model parameters (distribution of stroke severity, probability of large vessel occlusion as a function of stroke severity, ability to communicate as a function of stroke severity, estimated reduction in disability-adjusted life-years per minute faster treatment as a function of age, sex, stroke severity, and treatment modality [thrombolysis or mechanical thrombectomy], and average sleep duration) on maximally allowable annual cost of device (MAACD) according to age and annual risk of ischaemic stroke. **(A)** MAACD according to age and annual risk of ischaemic stroke (base-case socio-geographic scenario). Rows 1–6 show MAACD maps for the contra-device (left hand side) and pro-device (right hand side) values of model parameters indicated on the left. Row 7 shows MAACD maps for multiway sensitivity analyses including all parameters of row 1–6. **(B)** relative changes of MAACD associated with individual and joint changes of model parameters in socio-geographic scenarios I–VIII. For a definition of socio-geographic, see main text and Table 7. NIHSS stands for National Institutes of Health Stroke Scale, LVO for large vessel occlusion, ATC for ability to communicate, TE for treatment effect, ASD for average sleep duration, and WTP for willingness-to-pay threshold.

The impact of variation of model parameters (distribution of stroke severity, probability of LVO, probability of inability to communicate, distribution in reduction in DALYs per minute faster treatment, average sleep duration, and willingness-to-pay threshold) was investigated in one-way and multi-way sensitivity analyses. The results according to socio-geographic scenario are displayed in Figure 5. The largest MAACD changes observed in multiway sensitivity analyses are −57.1% and +162.0% in socio-geographic scenario IV and III, respectively.

### Illustration of results for specific populations

**Figure 6:**
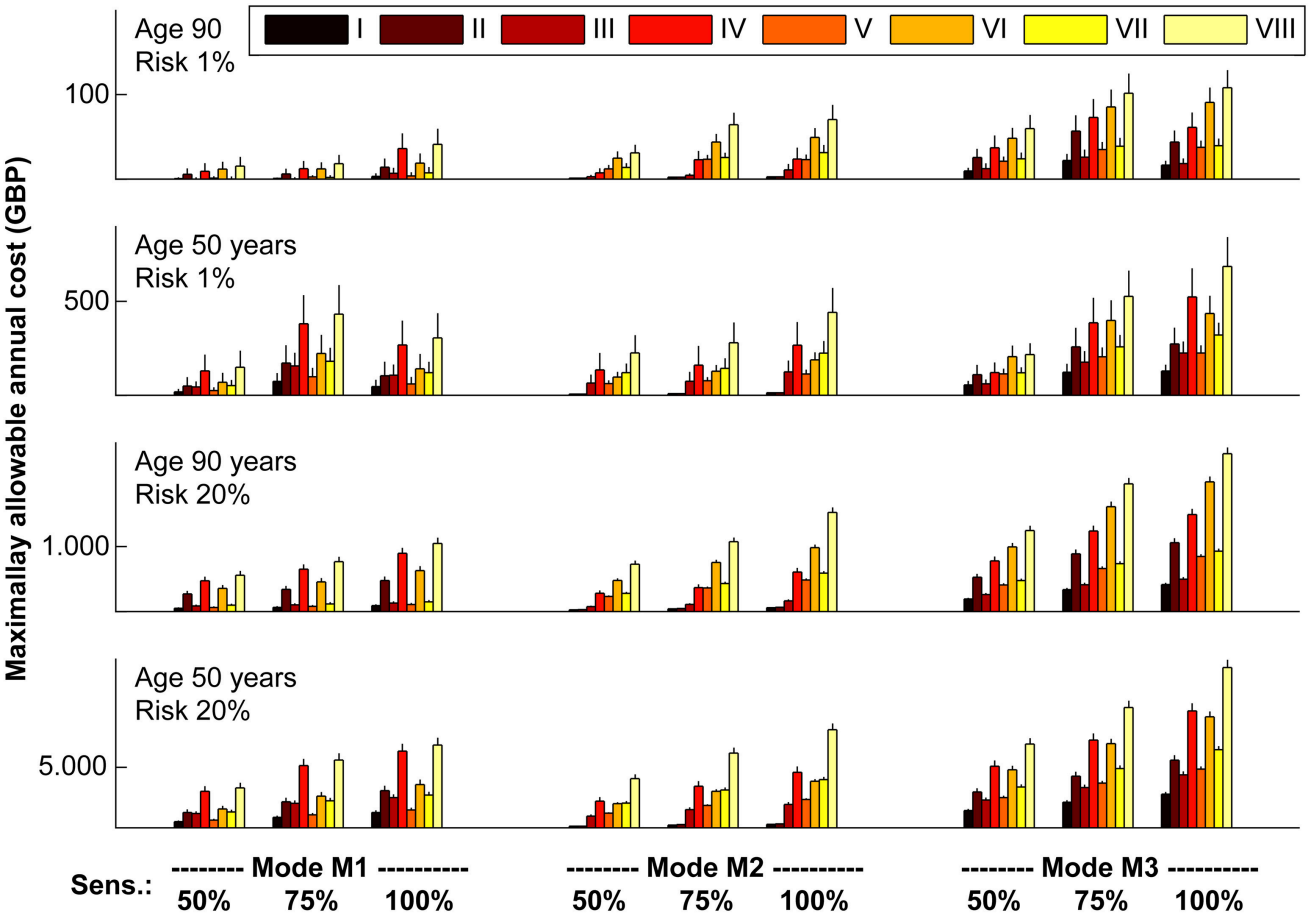
Maximally allowable annual cost of device according to socio-geographic scenario (I–VIII), mode of operation (M1–M3) and sensitivity (50, 75, and 100%) for acute ischaemic stroke detection in four demographically distinct sub-populations. Vertical lines represent standard errors of the mean. For a definition of socio-geographic scenarios and modes of operation of the device, see main text and Tables 4, 7. Note the different scaling of the vertical axes.

To illustrate our results, we calculated MAACD according to socio-geographic scenario, mode of operation and sensitivity for AIS detection for four demographically distinct populations (Figure 6). Consistent with the previous results, MAACD is highest in a population of 50 year old individuals with a high annual ischaemic stroke risk belonging to socio-geographic scenario VIII (rural environment, poor education with regards to stroke symptoms, long expected time found after incident/time last seen well before incident) under the assumption of an ischaemic stroke detection device with 100% sensitivity and detection of ischaemic stroke irrespective of vessel status and time of day (mode of operation M3). For these individuals, the MAACD is GBP 13,255.23 (95% confidence interval: GBP 12,607.58–13,902.89).

## Discussion

### Summary of findings

We used a probabilistic conditional analytical model and empirical parameter distributions derived from the literature to estimate the maximal annual cost that would render a real-time ischaemic stroke detection device a cost-effective health care intervention given currently accepted willingness-to-pay thresholds. We found that the MAACD varies significantly with age, annual risk of ischaemic stroke, and the social and geographic environment of the targeted individual. Even for young patients with an extremely high risk of ischaemic stroke (50 years old/20% risk) and living circumstances that would favor the use of a real-time ischaemic stroke detection device with 100% sensitivity, the upper limit for the annual cost would be GBP 29,449.10 including the uncertainty derived from multiway sensitivity analyses. For a population with more commonly encountered demographic and clinical characteristics (80 years old/1% annual risk of ischaemic stroke)(30), the MAACD in the base-case scenario would be only GBP 81.51.

### Availability of real-time stroke detection devices

To the best of our knowledge, there does not currently exist a device that would be able to continually monitor patients and to automatically detect an ischaemic stroke. However, with the ongoing technological improvements in the areas of miniaturization, decreased energy consumption, automated analysis of complex and large data sets, and man-machine interfaces, and our increased understanding of the pathogenesis and pathophysiology of ischaemic stroke, construction of such a device might be possible in the future. Based on the currently available technologies, methods employed could include implantable continuous Doppler ultrasound (31–33), motion analysis (34, 35), video surveillance, surface electromyography (36–39), or cerebral oximetry (40).

### Profitability of investments in real-time stroke detection devices

Our results suggest that for real-time AIS detection to be cost-effective in large group of unselected patients, devices would need to operate at a very low annual cost. Conversely, devices with higher annual cost would be cost-effective only in a very small group of selected patients at a young age with high risk of ischaemic stroke. Considering the large financial investments likely to be required to develop a real-time ischaemic stroke detection device and the expected magnitude of annual running costs given their likely technological complexity, it seems doubtful that investments in the development of functioning real-time AIS detection devices or indeed their sale and distribution / marketing in a public health insurance environment could be profitable.

### Study limitations

The following limitations of our study need to be considered when interpreting our results. First, we only considered a time-horizon of one single year, which allowed us to examine the annual running costs and the costs to set up the device together. This would not be appropriate for devices that require relatively high costs for installment and that could be used for several years, e.g., long-living electronical implants. Modelling of a longer time-horizon with separate accounting for setup costs and maintenance costs would require data on the evolution of annual risk of ischaemic stroke over time and on the relationship between risk for ischaemic stroke and mortality. In spite of this limitation, our results can be applied to hypothetical devices that are used over several years if the total costs that accrue over the whole time are expressed as appropriately discounted equivalent annual costs. Second, while we presented results for hypothetical devices with different modes of operation (detection independent of vessel status vs. detection limited to patients with LVO; and detection irrespective of time of day vs. detection limited to daytime when the patient would be active) and different sensitivities, we did not separately consider false positives, i.e., the incorrect detection of a stroke mimic or haemorrhagic stroke as AIS. However, due to the chosen characteristics of our target population, this does not lead to the introduction of bias: by excluding patients with known conditions that may produce stroke like symptoms such as epilepsy or migraine, patients in whom the device detects an ischaemic stroke incorrectly would be expected to receive prompt diagnostic work-up in the control scenario as well as in the intervention scenario, albeit with a greater delay. As no specific therapeutic interventions with a time-dependent effect-size exist for most stroke mimics and for haemorrhagic stroke not associated with the use of anticoagulants, costs and clinical outcome are expected to be similar in the control and intervention scenario, i.e. false positives do not contribute to incremental cost or benefit within the scope of the model. Third, it is conceivable that patients with transient stroke symptoms that would have resolved by the time the patients arrives at the hospital in the control scenario would be treated with thrombolysis or MT in the intervention scenario due to earlier evaluation by a stroke neurologist. In this case, early detection would be associated with additional costs for the acute treatment, but would not confer additional benefit. This possibility was not accounted for in our model due to lack of reliable information on the required parameters; as a consequence, our model may have overestimated MAACD which would not alter the interpretation of our findings. Last, we extracted most model parameters and distributions from the literature. However, for a subset of parameters, for example the sigmoid relationship between stroke severity and the ability to communicate, and the distributions of stroke severity used in sensitivity analyses, reasonable assumptions based on clinical experience had to be used. To ensure transparency, all parameters including their distributions and ranges used in sensitivity analyses are presented in the Online Data Supplement.

## Conclusion

In conclusion, we examined hypothetical devices to continually monitor at-risk patients for the occurrence of ischaemic stroke and present maximally allowable annual costs for these devices to operate in a cost-effective manner given a willingness-to-pay threshold of GBP 30,000.00. Our data suggest that such devices are expected to be cost-effective only for a small group of highly selected individuals.

## Data availability

All data for this study are included in the manuscript and the Online Data Supplement. The source code of the model is available upon request from LS.

## Author contributions

LS conceived the study, reviewed the literature, developed the model, performed the simulation, analyzed and interpreted the data, wrote the manuscript, and approved the final version of the manuscript.

### Conflict of interest statement

The author declares that the research was conducted in the absence of any commercial or financial relationships that could be construed as a potential conflict of interest.
