# Supplementary material for: Disability Adjusted Life Years due to Ischaemic Stroke Preventable by Real-Time Stroke Detection—A Cost-Utility Analysis of Hypothetical Stroke Detection Devices
Source: Front Neurol. 2018 Oct 1;9:814. doi: 10.3389/fneur.2018.00814 (PMC6174318; doi:10.3389/fneur.2018.00814)
Supplement: Supplementary file 1 [file Data_Sheet_1.PDF]

ONLINE SUPPLEMENT

Disability adjusted life years due to ischaemic stroke preventable by real-time stroke detection – a cost-utility analysis of hypothetical stroke detection devices

Ludwig Schlemm

Contents

1. Supplemental Figures 2

Figure S1. Delay due to poor education with regards to stroke symptoms as a function of symptom severity. 2

Figure S2. Probability distribution of symptom severity. 3

Figure S3. Probability of being able to communicate as a function of symptom severity. 4

Figure S4. Probability of large vessel occlusion as a function of symptom severity. 5

Figure S5. Indirect cost savings of mechanical thrombectomy vs. treatment with thrombolysis for patients with large vessel occlusion. 7

Figure S6. Probability distribution of time of ischaemic stroke occurrence. 8

1. Supplemental Figures

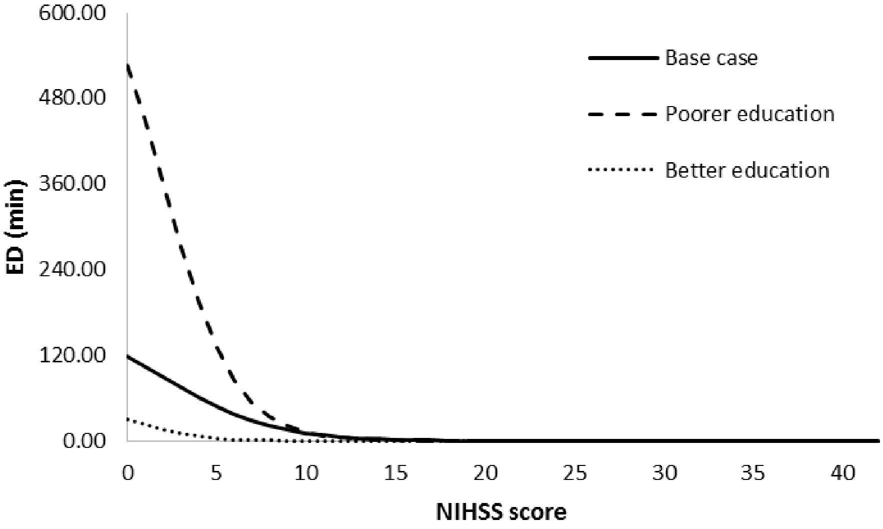

Figure S1. Delay due to poor education with regards to stroke symptoms as a function of symptom severity.

Expected delay between recognition of stroke symptoms and decision to seek medical attention in a thrombolysis-ready hospital (ED). It is assumed that patients with less severe strokes symptoms will wait longer before making the decision to go the a hospital than patients with more severe strokes symptoms. Distributions estimated from personal experience. Due to uncertainty of parameters, a wide range was used in sensitivity analyses. The following table shows the coefficients of the used sigmoid function. NIHSS stands for National Institutes of Health Stroke Scale.

**Scenario**	**Coefficients of the sigmoid function** $\boldsymbol{ED(NIHSS) = 60a \times \left(1 - \frac{1}{1 + e^{\frac{b - NIHSS}{c}}}\right)}$		
	**a**	**b**	**c**
Base-case	3	2	3
Poorer education (pro detection device)	12	2	2
Better education (contra detection device)	1	0	2

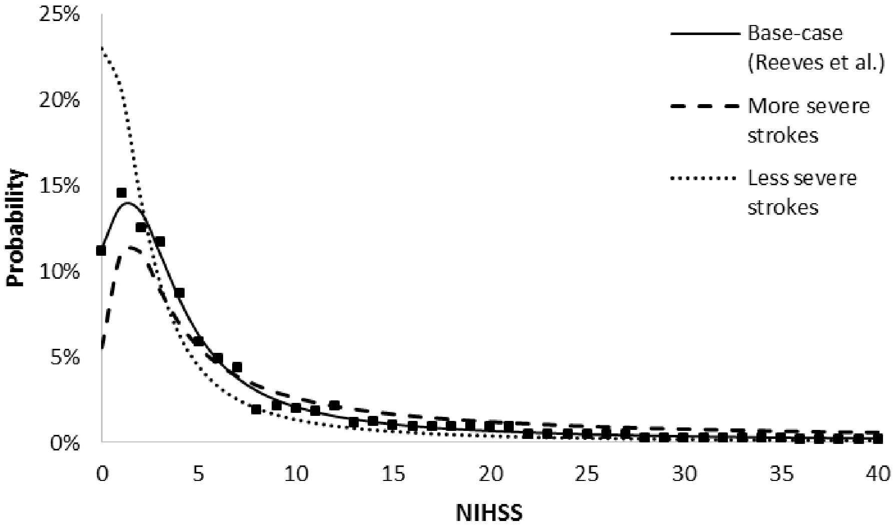

Figure S2. Probability distribution of symptom severity.

Symptom severity is measured by the National Institute of Health Stroke Scale (NIHSS), ranging from 0 – 42, with higher scores indicating more severe strokes. Data for the base-case scenario are extracted from Reeves et al. (1) and interpolated using the rational function $p(NIHSS) = \frac{p1 \times NIHSS + p2}{NIHSS^2 + q1 \times NIHSS + q2}$. Modified distributions with higher probabilities for respectively less severe and more severe strokes were created manually. The following table shows the coefficients of the interpolation function.

**Scenario**	**Coefficients of the sigmoid function** $p(NIHSS) = \frac{p1 \times NIHSS + p2}{NIHSS^2 + q1 \times NIHSS + q2}$ **(with 95% confidence bounds)**			
	**p1**	**p2**	**q1**	**q2**
Base-case, Reeves et al.(1) (median NIHSS 6.7)	0.0684 (0.0349, 0.102)	1.137 (0.8656, 1.409)	-2.288 (-2.676, -1.9)	10.02 (7.921, 12.12)
More severe strokes (median NIHSS 10.1; pro detection device)	1	1	-1	4
Less severe strokes (median NIHSS 4.4; contra detection device)	0.02	1	-0.3	5

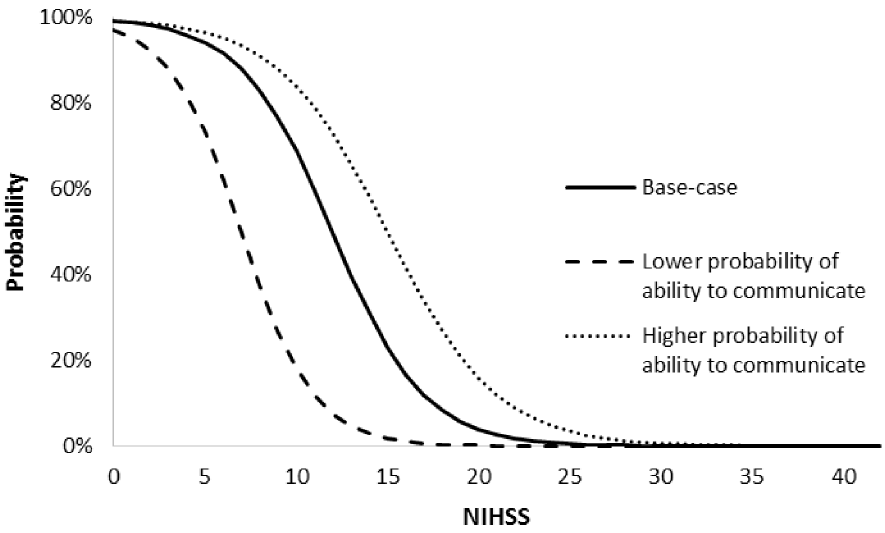

Figure S3. Probability of being able to communicate as a function of symptom severity. Inability to communicate defined as inability to contact emergency medical personnel independently and to give information about symptom onset due to stroke symptom severity (e.g. aphasia, motor impairment, reduced level of consciousness). It is assumed that patients with more severe strokes have a lower probability of being able to communicate. Distributions estimated from personal experience. Due to uncertainty of parameters, a wide range is used in sensitivity analyses. The following table shows the coefficients of the used sigmoid function. NIHSS stands for National Institutes of Health Stroke Scale.

**Scenario**	**Coefficients of the sigmoid function** $P_{ATC=1}(NIHSS) = 1 - \frac{1}{1+e^{\frac{a-NIHSS}{b}}}$	
	**a**	**b**
Base-case	12	2.5
Lower probability of being able to communicate (pro detection device)	7	2
Higher probability of being able to communicate (contra detection device)	15	3

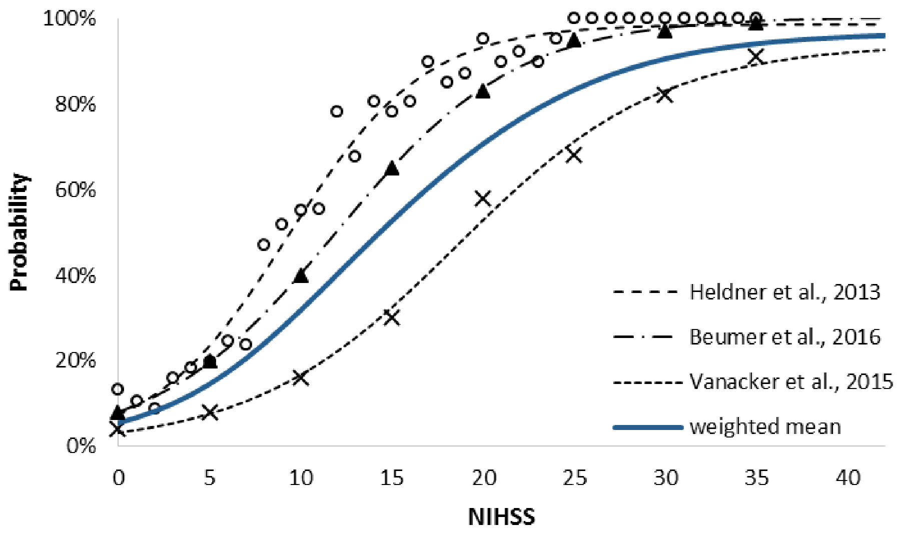

Figure S4. Probability of large vessel occlusion as a function of symptom severity. Data extracted from Heldner et al., (2) Vanacker et al., (3) and Beumer et al.(3) and interpolated using the sigmoid function $P_{LVO=1}(NIHSS) = \frac{a}{1+e^{\frac{b-NIHSS}{c}}}$. The following table shows the definition for 'large vessel occlusion' (LVO) used by the authors in their publications, and the coefficients of the sigmoid interpolation function. Since in comparison to contemporary eligibility criteria for endovascular therapy, these criteria are either too strict (Vanacker et al.) or too wide (Heldner et al. and Beumer et al.), we used the weighted mean of the three curves (with double weight for Vanacker et al.) in our base-case scenario, and the curves derived from Heldner et al. and Vanacker et al. as extreme values in our sensitivity analyses. NIHSS stands for National Institutes of Health Stroke Scale.

Author	Definition of LVO	**Coefficients of the sigmoid function** $P_{LVO=1}(NIHSS) = \frac{a}{1+e^{\frac{b-NIHSS}{c}}}$ **(with 95% confidence bounds)**		
		**a**	**b**	**c**
Heldner et al. (2) (pro detection device)	ICA, MCA (M1 or M2), basilar artery, intracranial VA	0.9853 (0.9551, 1.016)	9.359 (8.798, 9.92)	3.718 (3.216, 4.22)
Vanacker et al. (3) (contra detection device)	intracranial ICA (including the T), BA, MCA (M1)	0.9414 (0.8307, 1.052)	18.61 (16.43, 20.78)	5.619 (4.072, 7.165)
Beumer et al. (4)	intracranial ICA, anterior cerebral artery (A1), MCA (M1 or M2), intracranial VA, BA, PCA (P1 or P2)	1.002 (0.9852, 1.019)	1.98 (11.63, 12.34)	4.942 (4.64, 5.245)
Weighted mean		0.9596 (0.954, 0.9651)	14.12 (13.98, 14.26)	5.551 (5.431, 5.67)

ICA, internal carotid artery; BA, basilar artery; MCA, middle cerebral artery; VA, vertebral artery; PCA, posterior cerebral artery

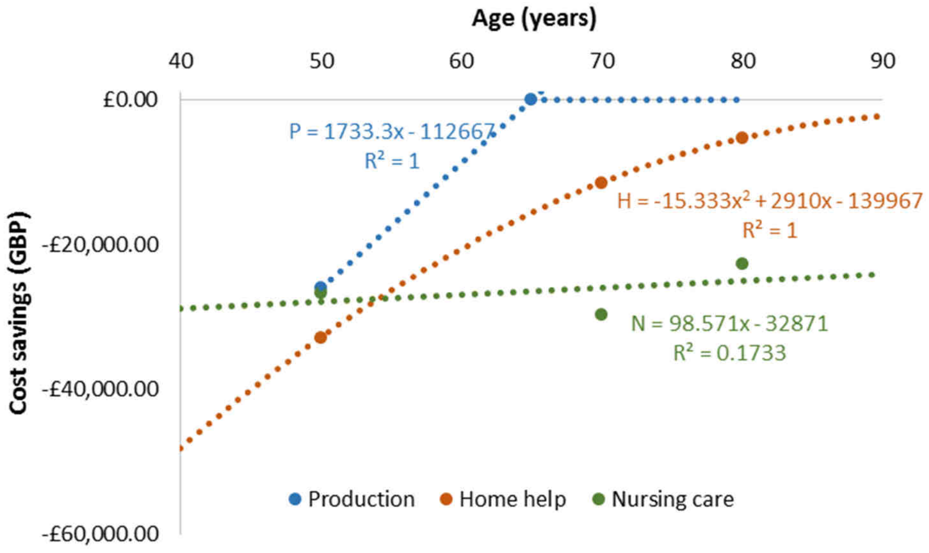

Figure S5. Indirect cost savings of mechanical thrombectomy vs. treatment with thrombolysis for patients with large vessel occlusion.

Data were extracted from Steen Carlsson et al. (5) and interpolated using linear or quadratic functions. Cost savings due to reduced loss of production are considered up to 65 years of age. Age-specific indirect cost savings correspond to a median NIHSS of 14, and a median time from symptom onset to reperfusion of approx. 241 min. (5) Assuming no clinical benefit and thus no cost-savings if reperfusion is achieved after 390 min, (maximum onset-to-groin puncture of 360 min + assumed groin to reperfusion time of 30 min), (6) we used linear transformations to obtain age-specific indirect cost savings per minute faster treatment. We then assumed a direct proportional relationship between the reduction in disability adjusted life-years per minute faster treatment reported by Meretoja et al. (7, 8) and the reduction in indirect costs per minute faster treatment. This is justified because both were calculated on the basis of functional outcome (modified Rankin scale) at three months. Using this assumption of proportionality, we obtained estimates for indirect cost-savings per minute faster treatment as a function of age, NIHSS, sex, and vessel status. Resulting cost savings were inflated at an annual rate of 3% to 2018 pounds sterling. NIHSS stands for National Institutes of Health Stroke Scale.

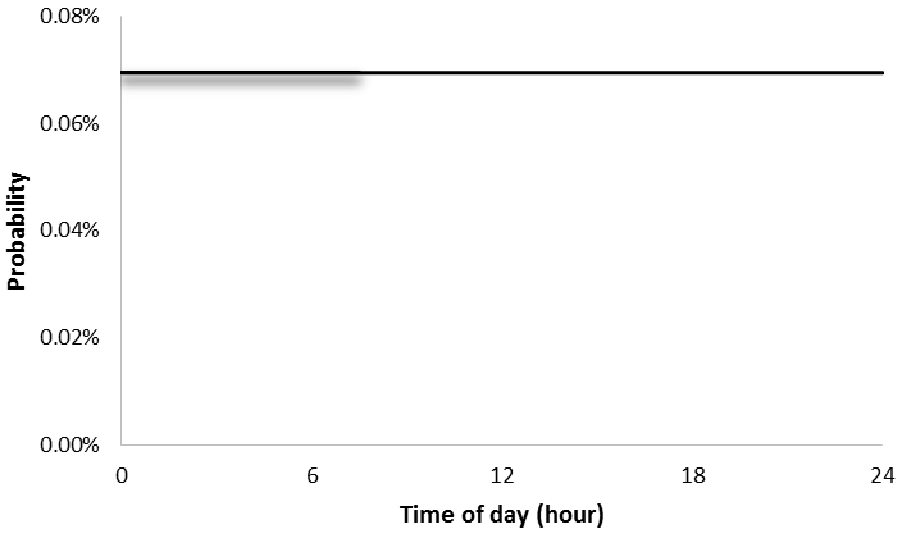

Figure S6. Probability distribution of time of ischaemic stroke occurrence.

With a granularity of 1 minute, the probability of time of ischaemic stroke occurrence for any given time of the day is equal to $\frac{1}{24\times 60} = 0.069\%$. The shaded area denotes the assumed average sleep duration (night time) in the base-case scenario (7.5 hours).

References

1. Reeves M, Khoury J, Alwell K, Moomaw C, Flaherty M, Woo D, et al. Distribution of National Institutes of Health stroke scale in the Cincinnati/Northern Kentucky Stroke Study. *Stroke* (2013) **44**(11):3211-3. doi: 10.1161/STROKEAHA.113.002881. PubMed PMID: 24003048; PubMed Central PMCID: PMCPMC4632977.
2. Heldner MR, Zubler C, Mattle HP, Schroth G, Weck A, Mono ML, et al. National Institutes of Health stroke scale score and vessel occlusion in 2152 patients with acute ischemic stroke. *Stroke* (2013) **44**(4):1153-7. doi: 10.1161/STROKEAHA.111.000604. PubMed PMID: 23471266.
3. Vanacker P, Heldner MR, Amiguet M, Faouzi M, Cras P, Ntaios G, et al. Prediction of Large Vessel Occlusions in Acute Stroke: National Institute of Health Stroke Scale Is Hard to Beat. *Crit Care Med* (2016) **44**(6):e336-43. doi: 10.1097/CCM.0000000000001630. PubMed PMID: 26958750.
4. Beumer D. Occurrence of intracranial large vessel occlusion in consecutive, non-referred patients with acute ischemic stroke. *Neurovascular Imaging* (2016).
5. Steen Carlsson K, Andsberg G, Petersson J, Norrving B. Long-term cost-effectiveness of thrombectomy for acute ischaemic stroke in real life: An analysis based on data from the Swedish Stroke Register (Riksstroke). *Int J Stroke* (2017) **12**(8):802-14. doi: 10.1177/1747493017701154. PubMed PMID: 28375069.
6. Powers WJ, Derdeyn CP, Biller J, Coffey CS, Hoh BL, Jauch EC, et al. 2015 American Heart Association/American Stroke Association Focused Update of the 2013 Guidelines for the Early Management of Patients With Acute Ischemic Stroke Regarding Endovascular Treatment: A Guideline for Healthcare Professionals From the American Heart Association/American Stroke Association. *Stroke* (2015) **46**(10):3020-35. doi: 10.1161/STR.0000000000000074. PubMed PMID: 26123479.
7. Meretoja A, Keshtkaran M, Saver JL, Tatlisumak T, Parsons MW, Kaste M, et al. Stroke thrombolysis: save a minute, save a day. *Stroke* (2014) **45**(4):1053-8. doi: 10.1161/STROKEAHA.113.002910. PubMed PMID: 24627114.
8. Meretoja A, Keshtkaran M, Tatlisumak T, Donnan GA, Churilov L. Endovascular therapy for ischemic stroke: Save a minute-save a week. *Neurology* (2017) **88**(22):2123-7. doi: 10.1212/WNL.0000000000003981. PubMed PMID: 28455382.
